# Week-to-week within-subject and between-subject biological variation of copeptin

**DOI:** 10.1515/cclm-2023-0673

**Published:** 2023-08-03

**Authors:** Nora Alicia Guldhaug, Eirik Åsen Røys, Kristin Viste, Per Medbøe Thorsby, Marit Sverresdotter Sylte, Janniche Torsvik, Heidi Strand, Bashir Alaour, Michael Marber, Torbjørn Omland, Kristin Moberg Aakre

**Affiliations:** Hormone Laboratory, Department of Medical Biochemistry and Pharmacology, Haukeland University Hospital, Bergen, Norway; Department of Medical Biochemistry and Pharmacology, Haukeland University Hospital, Bergen, Norway; Hormone Laboratory, Department of Medical Biochemistry and Biochemical Endocrinology and Metabolism Research Group, Oslo University Hospital, Aker, Oslo, Norway; Institute of Clinical Medicine and University of Oslo, Oslo, Norway; Multidisciplinary Laboratory Medicine and Medical Biochemistry, Akershus University Hospital, Lørenskog, Norway; King’s BHF Centre of Research Excellence, School of Cardiovascular Medicine and Sciences, King’s College London, London, UK; Department of Cardiology, Division of Medicine, Akershus University Hospital, Lørenskog, Norway; Department of Heart Disease, Haukeland University Hospital, Bergen, Norway; Department of Clinical Science, University of Bergen, Bergen, Norway

**Keywords:** analytical performance specifications, anti-diuretic hormone, biological variation, index of individuality, reference change values

To the Editor,

Copeptin is used as a sensitive surrogate marker of ADH (antidiuretic hormone) when investigating fluid imbalance such as in diabetes insipidus, SIADH (syndrome of inappropriate antidiuretic hormone secretion) and psychogenic polydipsia [1]. Unlike ADH, Copeptin concentration is stable in plasma [2]. Copeptin and ADH originate from the molecule pre-pro-vasopressin and are co-secreted in equimolar amounts. Plasma osmolality, hypovolemia and stress determine the secretion of both peptides from the neurohypophysis. Before implementation of high sensitivity cardiac troponin copeptin was suggested as an acute chest pain biomarker [2], and has recently evolved as a promising stroke marker [3].

Understanding the biological variation (BV) of copeptin and its components within-subject (CV_I_) and between-subject (CV_G_) variation has several applications, including the interpretation of test results, suggesting analytical performance specifications, determining sample size for steady-state concentration, calculating the reference change value (RCV) and the index of individuality (II). RCV is used to assess the significance of a change in serial measurements, whereas II is useful for determining whether the change in serial measurements rather than the reference intervals should be used to evaluate if the patient could be in a physiological vs. pathological status.

The European Federation of Laboratory Medicine (EFLM) variation database provides BV data and a checklist for studies estimating BV [4]. EFLM recommend BV estimations using ANOVA analysis with outlier exclusion [4], and the Bayesian approach for the estimation of BV was recently suggested [5]. This study aims to establish the week-to-week biological variation of copeptin using both ANOVA and the Bayesian methods.

This study was performed in accordance with the Declaration of Helsinki (REC ID number 2018/92 for Bergen and Oslo and South Central – Berkshire Research Ethics Committee for London) and EFLM checklist for biological variation studies (BIVAC) [4] and has been described earlier [6, 7]. A total of 30 presumably healthy volunteers were recruited from three different centers. The age range of the participants were 21–64 years (mean 38 years), and 8 of 16 women were presumed fertile. Weekly venous blood samples were collected for ten consecutive weeks, plasma was frozen at −80 °C and later analyzed for copeptin in one run at the Hormone Laboratory at Oslo University Hospital using a compact PLUS Copeptin proAVP Kryptor Kit (Brahms Kryptor, Thermo Fisher Scientific). The limit of detection (LoD) was 0.69 pmol/L, limit of quantification (LoQ) 1.1 pmol/L and long-term analytical variation (CV_A_) was 7 % at 5.3 pmol/L and 4 % at 99 pmol/L. The method is accredited according to NS-EN ISO/IEC 17025:2017, and used for analyzing copeptin concentrations reported to clinical care at Oslo University Hospital.

Statistical analysis including the detection and exclusion of outliers, checking for trends in the concentration, transforming skewed data, checking for homogeneity, and AVOVA were performed as per the BIVAC recommendations [4], and were described in detail in our previous work [6, 7].

The Bayesian model was applied as described in detail by Røraas et al. [5] which assumes Student t-distributions and accommodates extreme observations and non-homogeneous variances. In brief, the model infers a posterior distribution of CV_I_ (CV_P(I)_) with estimates of the mean (µCV_P(i)_) and SD (σCV_P(i)_). Based on these parameters the model provides a predicted distribution (dCV_P(I)_) based on randomly generated CV_P(i)_. The model also allows for assessment of heterogeneity through the Harris–Brown ratio [5, 8, 9], for both the estimated and predicted distribution of CV_I_. Ratios <100 %/sqrt (2S), where S is the average number of samples per individual, would signal a homogenous population, and for our study S would be 10, so <22.4 %. The prior assumptions used in this model were based on our ANOVA results, where we applied the following prior distributions and hyperparameters, N-truncated indicates that only the positive part of the normal distribution is used in the estimation routine, and SDs are defined as positive, the 10 % (0.1) of the SD has been applied to the hyperparameters):

$${SD}_{P\left(i\right)}∼\;N_{truncated}\left\{\mu \left[{SD}_{P\left(i\right)}\right],\sigma \left({SD}_{P\left(i\right)}\right]\right\}$$

$$\sigma \left[{SD}_{P\left(i\right)}\right]∼N_{truncated}\left(0,0.1\right)$$

$$\mu \left[{SD}_{P\left(i\right)}\right]∼N_{truncated}\left({SD}_{I},0.1\times {SD}_{I}\right)$$

$${SD}_{G}∼N_{truncated}\left({SD}_{A},0.1\times {SD}_{G}\right)$$

$${SD}_{A}∼N_{truncated}\left({SD}_{A},0.1\times {SD}_{A}\right)$$

The asymmetrical RCV values (with 95 % confidence intervals) were calculated according to Fokkema et al. [8]:

$${RCV}_{pos}\;\left[\exp\left(1.96\times 2\;2^{\frac{1}{2}}\;x\;{\left(\sigma_{A}^{2}+\sigma_{I}^{2}\right)}^{\frac{1}{2}}\right)-1\right]\times 100$$

$${RCV}_{neg}=\left[\exp(-1.96\times 2^{\frac{1}{2}}\times {\left(\sigma_{A}^{2}+\sigma_{I}^{2}\right)}^{\frac{1}{2}})-1\right]\times 100$$

in which σ_A_ is the analytic standard deviation and σ_I_ is the within-person standard deviation of the logarithmic data.

The II was calculated using the retransformed data as follows [2]:

$$II=\frac{\sqrt{CV\frac{2}{A}+CV\frac{2}{I}}}{{CV}_{G}}$$

Desirable analytical performance specifications were calculated as [2]:

$${CV}_{A}<\frac{1}{2}{CV}_{I}$$

$$Bias<\frac{1}{4}\sqrt{{CV}_{I}^{2}+CV\frac{2}{G}}$$

Two specialists in endocrine biochemistry (KMA and KV) classified females into fertile and peri/postmenopausal groups based on the concentration and covariation observed in FHS, LH, estradiol and progesterone during the 10-week data collection period (weekly samples during 2.5 menstrual cycles) of the study.

**Figure 1:**
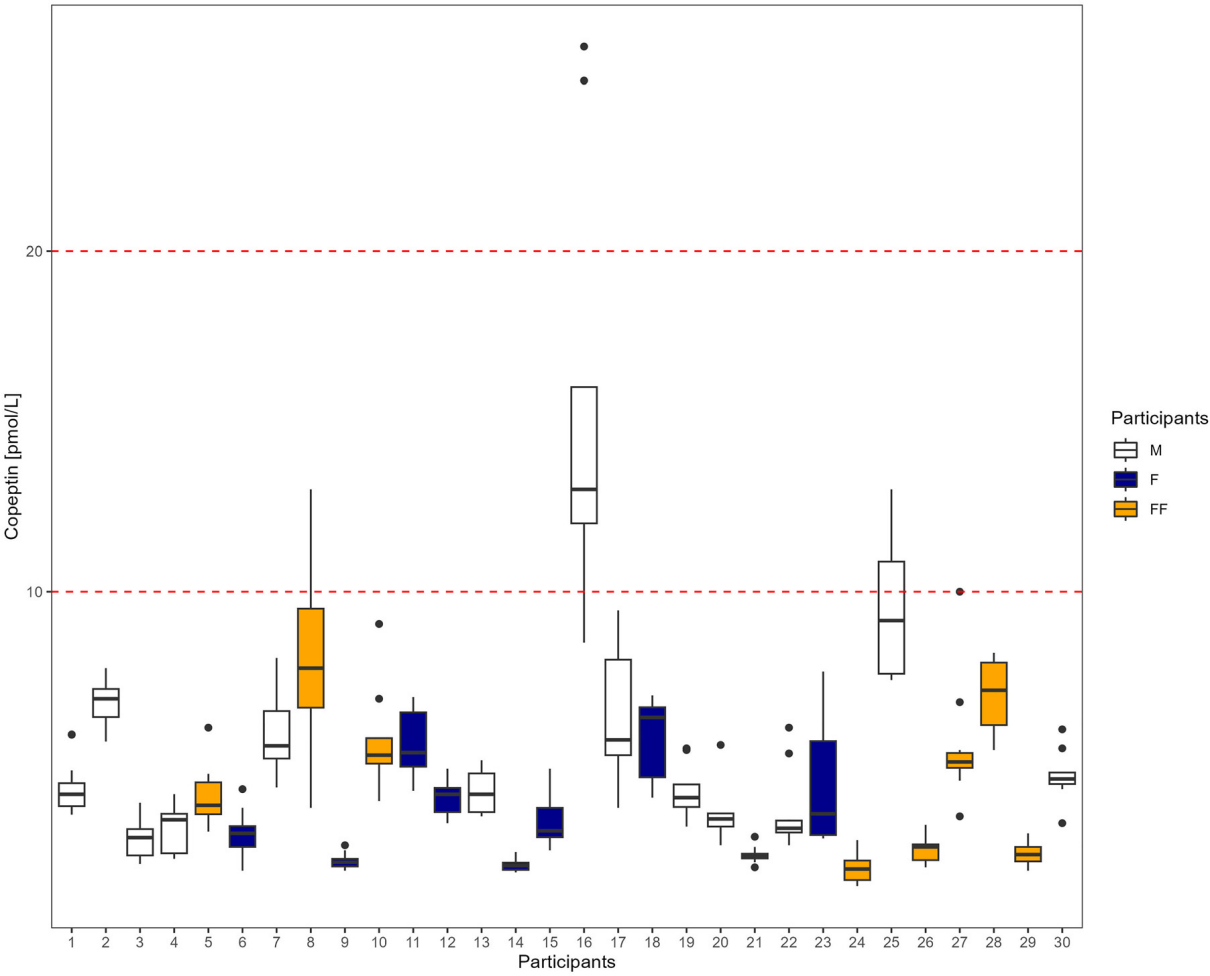
Median, 25 to 75 percentile and total range of copeptin concentrations by participants. ID 8, 16, 23 were excluded as outliers before the CV-ANOVA analysis. M, male; F, peri/postmenopausal female; FF, fertile female.

The distribution of the concentrations of the 30 participants are shown in Figure 1. Three subjects were identified as outliers (Table 1) (based on trend, Reeds criterion and non-homogeneity) and were not included in the ANOVA analysis. For the Bayesian approach estimates, no outliers needed to be excluded.

**Table 1:** Biological variation, RCV and II are presented in the table. The top rows show the number of outliers subjects and samples included in the analysis of analytical-(CV_A_), within (CV_I_) and between subject biological-variation (CV_G_). Uncertainty is estimated as 95 % confidence/credibility-intervals (C(r)I) as applicable.

	CV-ANOVA				Bayesian approach			
Subgroup	Total	Male	Female	FF	Total	Male	Female	FF
Outliers (samples): analytical	2		1	1	2		1	1
Outliers (individ): trend	1		1	1				
Outliers (individ): Reed test	1	1						
Outliers (individ): homogeneity	1		1					
Samples/participants	268/27	130/13	139/14	69/7	292/30	138/14	156/16	77/8
Median concentration (25–75 percentiles)	3.7 (2.6–5.4)	4.1 (3.2–5.6)	3.3 (2.2–5.0)	3.5 (2.3–5.5)	3.9 (2.7–5.8)	4.2 (3.2–6.3)	3.6 (2.4–5.3)	3.9 (2.5–6.1)
CV_A_ (95 % CI/CrI)^a^	6.9 (6.3–7.5)	7.6 (6.8–8.6)	6.6 (5.7–8.0)	6.2 (5.5–7.1)	6.7 (5.9–7.7)	6.0 (4.8–7.4)	7.3 (6.3–8.3)	5.9 (5.0–5.8)
CV_I_ (95 % CI/CrI)^a^	19.8 (17.9–21.8)	19.3 (16.9–22.2)	21.0 (17.4–25.6)	20.2 (17.7–23.4)	19.5 (17.0–22.0)	20.5 (16.3–24.8)	19.4 (16.6–22.2)	19.4 (16.2–22.7)
dCV_P(I)_ median (20–80 percentile)	N/A	N/A	N/A	N/A	20.9 (15.4–25.5)	21.4 (16.7–26.2)	20.2 (14.5–26.2)	21.5 (16.7–26.9)
CV_G_ (95 % CI/CrI)^a^	44.9 (34.3–64.3)	47.3 (33.1–83.4)	54.7 (33.1–159.7)	40.9 (28.3–73.0)	47.3 (39.6–55.0)	48.4 (34.2–62.6)	53.7 (14.5–26.2)	43.1 (35.2–50.9)
RCV_pos_. mean (95 % CI)	77.7 (69.7–87.2)	78.6 (67.7–93.8)	76.9 (66.4–90.4)	83.0 (67.1–106.0)	76.2 (65.4–87.9)	79.8 (61.4–101)	76.8 (64.8–90.0)	74.6 (60.8–90.1)
RCV_neg_. mean (95 % CI)	−43.7 (−41.1 to −46.6)	−44.0 (−48.4 to −40.4)	−43.5 (−48.0 to −40.0)	−45.4 (−52.0 to −40.1)	−43.3 (−39.5 to −46.8)	−44.4 (−38.0 to 50.2)	−43.4 (−39.3 to −47.3)	−42.7 (−37.8 to −47.4)
II	0.48	0.50	0.46	0.40	0.44	0.40	0.39	0.47
Estimated Harris–Brown ratio	N/A	N/A	N/A	N/A	27	26	33	22
Predicted Harris–Brown ratio	N/A	N/A	N/A	N/A	30	32	37	34

N/A, not applicable; FF, presumed fertile females, ^a^results from Bayesian approach is presented with 95 % credibility interval.

Median copeptin concentration for all participants (30 individuals) was 3.9 (25 and 75 percentile, 2.7–5.8) pmol/L (Table 1). Analytical and biological variation, RCV and II for the total cohort and fertility-stratified sub-groups, using both methods are reported in Table 1. The estimation of the CV_I_ by the ANOVA and Bayesian approaches (μCV_P(I)_) produced similar results, with 2 percentage points differences or less. The RCV ranged (mean values) from approximately −40 % (deteriorating values) to 80 % (increasing values). The II was generally low (≤0.5). There were no major differences in the estimated parameters between the sub-groups, except a slightly higher CV_G_ and a slightly lower II in the fertile females. Both the estimated (reflecting the actual study data) and predicted (reflecting the simulated distribution) Harris–Brown ratios were generally high, indicating a within-subject heterogeneity in the distribution of copeptin concentrations in healthy individuals. Based on the data the following analytical performance specifications could be calculated; 9.9 % as desirable CV_A_ and 12 % as desirable analytical bias.

The main limitation in our study is the relatively low number of included subjects, however all estimates are given with confidence intervals indicating the level of uncertainty. The subgroups were small, resulting in larger uncertainty for the specific estimates, and these need confirmation in other studies. The fertility status were determined based on expert consensus. We did not collect data on menstrual cycles so we could not determine if copeptin varied according to the menstrual status. Also, our study only included healthy subjects, so the biological variation data are not applicable for interpreting clinically relevant changes in patients with chronic disease.

To our knowledge weekly biological variation of copeptin has not been reported in previous studies. Our data indicate that important sex differences in the biological variation of copeptin are unlikely. Further, no definitive differences between fertile women and postmenopausal women or men were detected. Based on the low II (<0.6), delta values should be preferred for identifying a possible clinical change as compared to reference intervals. During physiological conditions delta values between two serial measurements could range from −40 to 80 %. As the Harris–Brown ratio indicated heterogeneity for all subgroups, a strategy of using different dCV_p(i)_ percentiles as an alternative to the mean CV_I_ may be adopted to set analytical performance specifications or calculate RCV, as suggested by Aarsand et al. [9], depending on the local clinical needs. Finally, the results indicate that routine laboratories have the potential to achieve satisfactory analytical performance when copeptin is measured, given the significant disparity between biological and analytical variation.
